# Balanced crystalloids versus isotonic saline in critically ill patients: systematic review and meta-analysis

**DOI:** 10.1186/s40560-018-0320-x

**Published:** 2018-08-17

**Authors:** Yazan Z. M. Zayed, Ahmed M. Y. Aburahma, Mahmoud O. Barbarawi, Kewan Hamid, Momen R. N. Banifadel, Laith Rashdan, Ghassan I. Bachuwa

**Affiliations:** 1 Internal Medicine Department, Hurley Medical Center/Michigan State University College of Human Medicine, Flint, MI USA; 2 Internal Medicine Department, University of Toledo College of Medicine and Life Sciences, Toledo, OH USA; 3 East Lansing, USA

**Keywords:** Balanced crystalloids, Isotonic saline, Critically ill patients, Fluid therapy and meta-analysis

## Abstract

**Objectives:**

Intravenous fluids are one of the most used medical therapy for patients, especially critically ill patients. We conducted a meta-analysis comparing between balanced crystalloids and normal saline in critically ill patients and its effect on various clinical outcomes.

**Design:**

Meta-analysis and systematic review of randomized clinical trials (RCTs).

**Methods and data source:**

Electronic search was performed using PubMed, Cochrane library, and clinical trials.gov from inception through March 1, 2018, with inclusion of prospective studies that investigated one of the primary outcomes which were acute kidney injury (AKI) and in-hospital mortality while secondary outcomes were intensive care unit (ICU) mortality and new renal replacement therapy (RRT).

**Results:**

Six RCTs were included. Total of 19,332 patients were included in the final analysis. There was no significant difference in in-hospital mortality (11.5% vs 12.2%; OR 0.92; 95% CI 0.85–1.01; *P* = 0.09; *I*^2^ = 0%), incidence of AKI (12% vs 12.7%, OR 0.92; 95% CI 0.84–1.01; *P* = 0.1; *I*^2^ = 0), overall ICU mortality (OR 0.9, 95% CI 0.81–1.01, *P* = 0.08, *I*^2^ = 0%), or need for new RRT (OR 0.92, 95% CI 0.67–1.28, *P* = 0.65, *I*^2^ = 38%) between balanced crystalloids and isotonic saline in critically ill patients.

**Conclusion:**

Balanced crystalloids and isotonic saline have no difference on various clinical outcomes including in-hospital mortality, AKI, overall ICU mortality, and new RRT. Further powerful clinical trials are required to determine the relationship between crystalloid fluid type and clinical outcomes.

**Electronic supplementary material:**

The online version of this article (10.1186/s40560-018-0320-x) contains supplementary material, which is available to authorized users.

## Background

Intravenous fluids are one of the most commonly used medical therapies for patients especially in intensive care units (ICUs). Isotonic saline has been the most commonly used crystalloid for fluid resuscitation [1, 2].

The balanced crystalloids like lactated Ringer’s and Plasma-Lyte solutions have an electrolyte composition which is closer to plasma. Balanced crystalloids are increasingly used for resuscitation of patients undergoing surgery, trauma, and diabetic ketoacidosis especially with data suggesting that the use of isotonic normal saline is associated with increased risk of acute kidney injury (AKI) and hyperchloremic metabolic acidosis [3–8]. On the other hand, some of the balanced solutions are considered hypotonic given the lower sodium concentrations and are associated with metabolic alkalosis, hyperlactemia, and hypotonicity especially when administered in large volumes [2].

Many studies have investigated the effect of crystalloid fluid type given for patients and adverse outcomes. In a prospective study in critically ill patients, chloride-restrictive fluid strategy was associated with decreased incidence of AKI and need for new renal replacement therapy (RRT) when compared to rich chloride fluids [3]. In addition, resuscitation with Plasma-Lyte solution at the day of surgery was associated with a decrease in rate of major complications including new RRT, incidence of infections, and blood transfusion in comparison to isotonic saline in a retrospective matched observational study [8].

Other prospective and observational studies have controversial findings as some studies showed that balanced solutions are associated with decreased rate of AKI, new RRT, and death [3, 5, 8, 9], while other studies found no difference in these outcomes between both strategies [10, 11].

A recent meta-analysis concluded that balanced fluids are more beneficial than isotonic saline in keeping postoperative electrolytes and acid-base balance among adult patients undergoing non-renal surgery [12]. On the other hand, a meta-analysis comparing between balanced and isotonic saline in operation rooms and ICUs showed no difference in AKI neither in-hospital mortality between the two fluid types [13].

In front of this conflict, we are performing a meta-analysis that involved all prospective trials comparing between balanced crystalloids and normal saline fluid resuscitation exclusively in critically ill patients. Balanced fluids include lactated Ringer’s, Hartmann’s solution, or Plasma-Lyte solutions while isotonic fluids pointed toward use of normal saline.

## Methodology

### Literature search and data source

We conducted our meta-analysis according to the Preferred Reporting Items for Systematic Reviews and Meta-Analyses Protocols (PRISMA-P) Statement 2015 [14]. An electronic literature search was performed independently and separately by three investigators (A.A., M. B., and Y.Z) in accordance with the recommendations of the Cochrane Collaboration, using PubMed, ClinicalTrials.gov, and Cochrane library from inception through March 01, 2018. Any disagreement was solved by a discussion of three reviewers and a fourth investigator (M.Ba.). Neither language nor demographic restrictions were applied. As well, references of the relevant studies and meta-analyses were reviewed for possible eligibility.

**Fig. 1:**
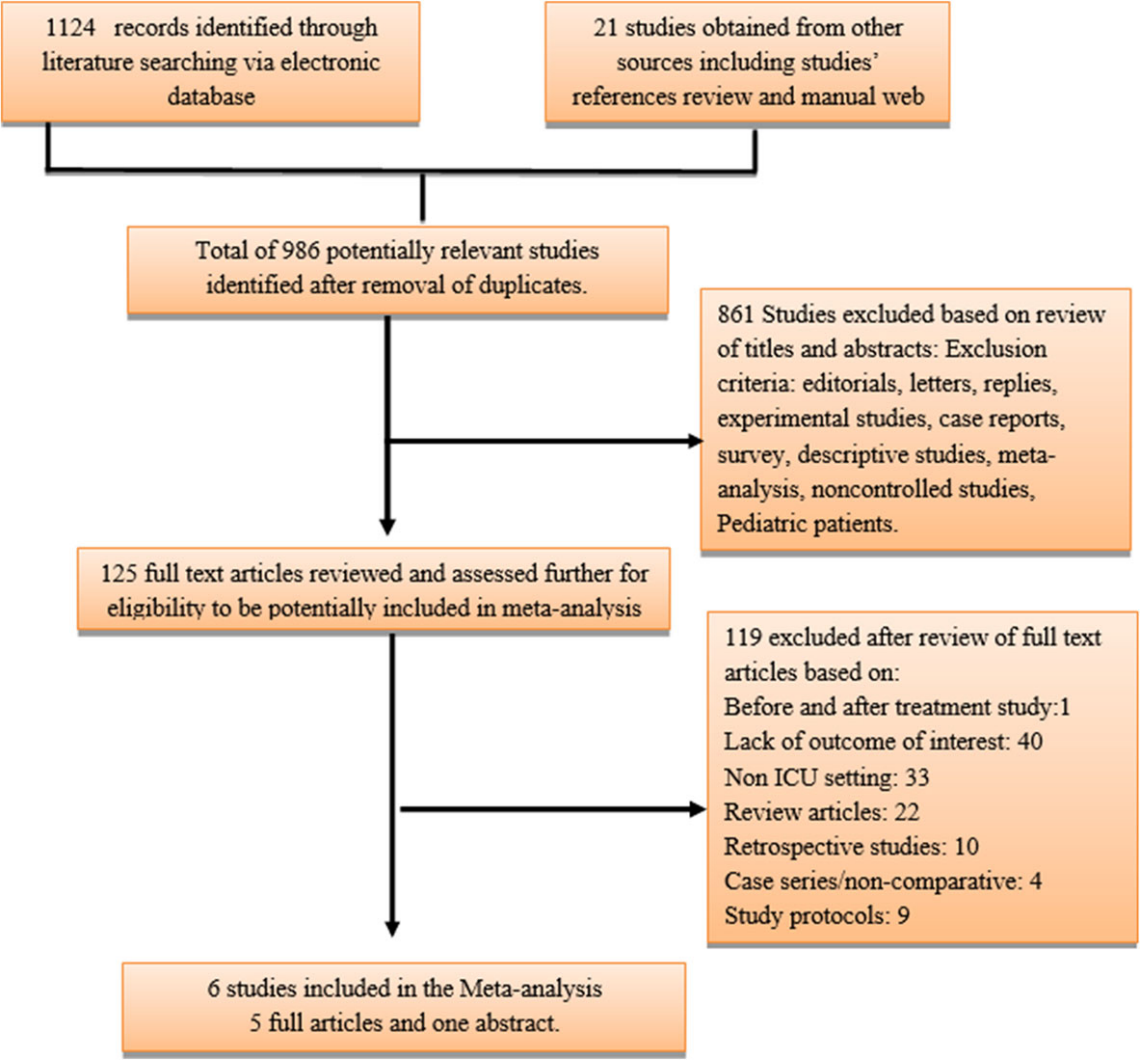
Flow diagram of literature search and study selection based on Preferred Reporting Items for Systematic Reviews and Meta-Analyses Protocols (PRISMA-P) recommendation

Studies were first screened by titles and abstracts for eligibility. The full texts of eligible studies were reviewed in the second step before exclusion. The search process is detailed in Fig. 1. The electronic search was archived through Mendeley program and is available on request.

### Study selection

We have included randomized clinical trials (RCTs) comparing between balanced crystalloids and isotonic saline in critically ill adult patients who received their management in ICUs. Studies that provided at least one of our primary endpoints which are in-hospital mortality and incidence of AKI were included. Retrospective studies and before andafter treatment studies were excluded to decrease bias and confounding variables. Of the prospective studies, we excluded all studies that investigated the effect of crystalloid fluid type given in non-ICU setting like emergency department or intra-operative rooms or given to non-critically ill patients.

**Fig. 2:**
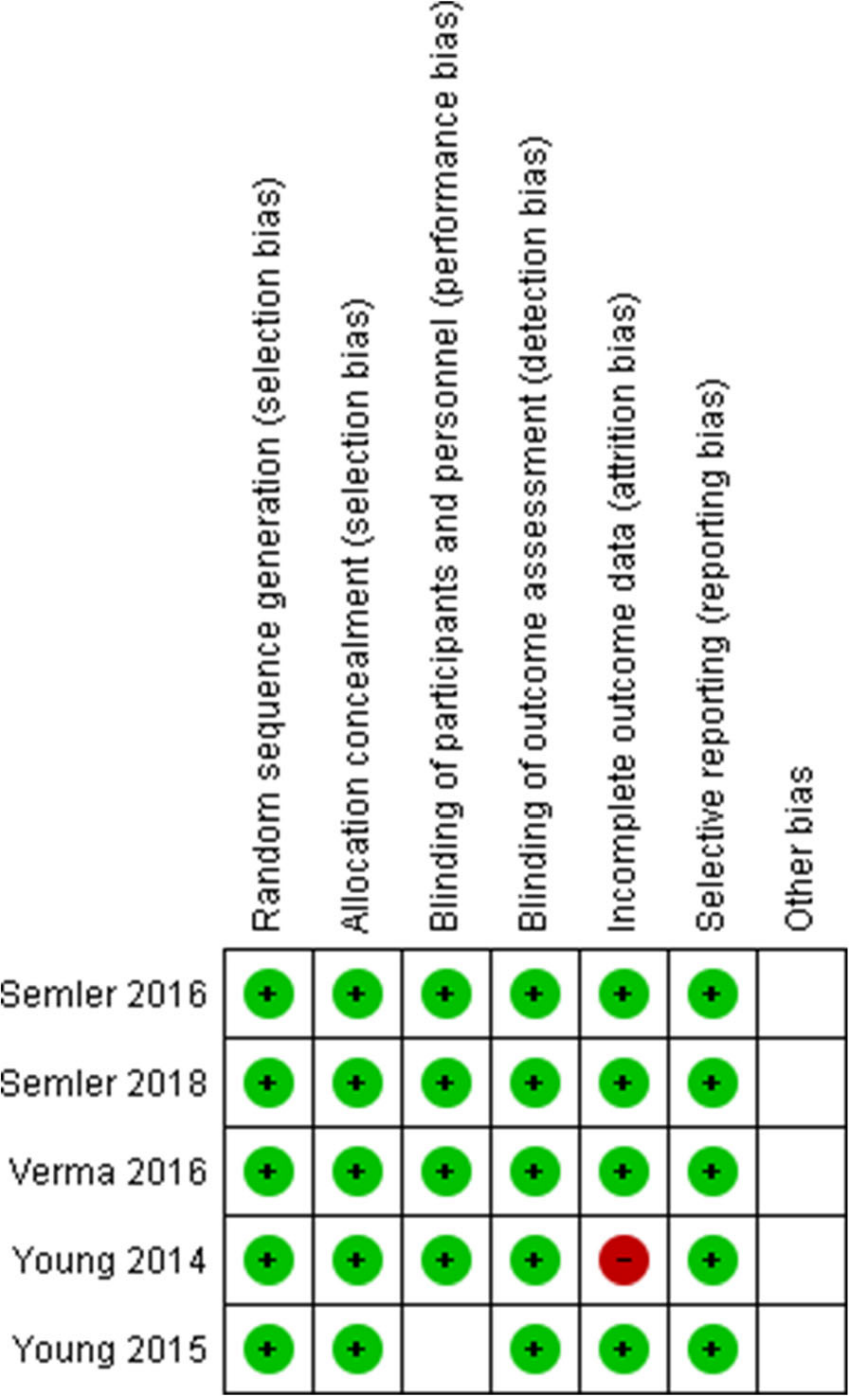
Risk of bias summary: review author’s judgments about each risk of bias item for each included study except Ratanarat et al.’s study

We performed quality assessment for the included RCTs for which we have full articles based on Cochrane collaboration’s tool for assessing risk of bias in RCTs. We assessed included RCTs for random sequence generation, allocation concealment, blinding of participant and personnel, blinding of outcome assessment, incomplete outcome data, selective reporting, and other biases. We classified studies as low risk for bias only if all the described items were adequately described as low risk. Quality assessment results are shown in Fig. 2.

### Data extraction

Three reviewers (A.A, M. B, Y.Z.) independently and separately extracted the data into a predesigned form from the included studies. Any disagreement was solved by a discussion between the three reviewers and fourth investigator (M.Ba.).

### Outcomes

The primary outcome measures are AKI and in-hospital mortality at the longest follow-up provided by the study for either outcome measures. Patient who developed stage 2 or higher based on KDIGO (Kidney Disease Improving Global Outcome) criteria or injury or higher based on RIFLE (Risk, Injury, Failure, Loss, End stage) criteria were included in for analysis of AKI. This was chosen to make the measurement of AKI outcome similar between studies which used either of those two cut-off points. As well, it represents moderate to severe kidney injury. In-hospital mortality is defined as mortality before discharge or at the longest follow-up period provided by the study.

Secondary outcomes were needed for new RRT and ICU mortality.

### Statistical analysis

Statistical analysis was performed using the comprehensive meta-analysis program. Statistics were pooled using random effect model; odds ratios (OR) for binary outcomes, with 95% confidence intervals (95% CI) were calculated. We measured two-sided *P* values for each outcome and statistical significance was determined by a two-tailed *P* value < 0.05. Heterogeneity among studies is reported using the *I*^2^, *Q* value statistic, and was gathered using random effect model for dichotomous co-variables. Since no enough data provided by the included studies about the outcomes in specific clinical situation like septic shock or surgical patients, sensitivity analysis could not be done.

## Results

### Search process and summary of included studies

A through literature search has resulted in 1124 articles from electronic searches and 21 articles from other sources including manual web search and references review of relevant studies and meta-analyses.

We have included six RCTs that compared fluid therapy with balanced crystalloids vs isotonic saline in critically ill patients [10, 11, 15–18]. Table 1 explains the summary of included studies and Fig. 1 showed the information relevant to the search process. Forty studies were excluded due to lack of outcome of interest as most of those studies were concerned about acid-base status change, effects on electrolytes, inflammatory markers, and fluid balance during first days after administration of intravenous fluids.

**Table 1:** Summary of included studies

First author	Year	Study design	Follow-up period for outcomes	Reported outcomes	Fluid type	Number of total patients
Young	2015	Multi-center double-blind, cluster randomized, double-crossover trial	90 days after randomizations for all variables	Hospital and ICU mortality, AKI and RRT	Plasma-Lyte	1152
					Saline	1110
Verma	2016	Multi-center double-bind randomized controlled trial	AKI during the first 4 days while other outcomes till discharge	Hospital and ICU mortality, AKI and RRT	Plasma-Lyte	33
					Saline	34
Semler (SMART trial)	2018	Single center unblinded, cluster randomized, multiple crossover trial	Death at 60 days, AKI after enrollment, RRT at 28 days.	Hospital and ICU mortality, AKI and RRT	LR or Plasma-Lyte	7942
					Saline	7860
Semler (SALT trial)	2016	Single-center prospective, open-label, cluster-randomized, multiple crossover trial	Death at 60 days, AKI after enrollment, RRT at 28 days.	Hospital and ICU mortality, AKI and RRT	LR or Plasma-Lyte	520
					Saline	454
Young	2014	Single center randomized, double-blind, parallel-group clinical trial	In-hospital mortality at 30 days	Hospital mortality	Plasma-Lyte	22
					Saline	24
Ratanarat	2017	Single-center randomized controlled trial	AKI during the first 7 days	AKI and RRT	Balanced	88
					Saline	93

*AKI* acute kidney injury, *RRT* renal replacement therapy, *ICU* intensive care unit, *LR* lactated Ringer’s

The total number of included patients in our review and meta-analysis is 19,332 patients, 9757 patients in balanced crystalloids group and 9575 patients in isotonic saline. A total of 19,151 patients were included in in-hospital mortality analysis and 18,337 patients were included in AKI and RRT analyses while ICU mortality analysis included 19,105 patients.

### Baseline characteristics of patients

We included a total of 19,332 patients in both groups, and a total of 12,066 out of 19,151 were male (57.7% in balanced fluids and 58.9% in isotonic saline). Sepsis and/or septic shock were diagnosed in 2709 out 19,151 (13.9% in balanced fluids and 14.3% in isotonic saline). Of the patients, 6647 out of 19,151 were on mechanical ventilator (38.1% in balanced fluid vs 31.2% in saline group).

Table 2 shows the demographic and baseline clinical characteristics of included study population.

**Table 2:** Baseline characteristics of study population

Study Name	Fluid type	Age (years)		Gender		CKD	Baseline serum creatinine (mg/dl)		Sepsis and/or septic shock	MV	Vasopressor	ICU admission indication	
		Mean (SD)	Median (IQR)	Male	Female		Mean	Median				Medical	Surgical
Young [10]	Balanced	60.1 (16.79)	–	64%	36%	–	0.98 (0.76)		4%	67%	–	29%	71%
	Saline	60.95 (16.25)		67%	34%		0.99 (0.68)		4%	66%		28%	72%
Verma [16]	Balanced	–	62 (45–70)	62%	38%	–	–	0.85 (0.58–1.34)	46%	58%	46%	58%	42%
	Saline		64 (46–72)	64%	36%			0.9 (0.6–1.21)	41%	56%	32%	48%	52%
Semler [15]	Balanced	–	58 (44–69)	57%	43%	17%	–	0.89 (0.47–1.1)	15%	34%	26%	78%	22%
	Saline		58 (44–69)	58%	42%	17%		0.89 (0.47–1.1)	15%	35%	26%	79%	21%
Semler [11]	Balanced	–	57 (44–68)	52%	48%	23%	–	–	25%	34%	22%	–	–
	Saline		58 (46–70)	54%	46%	23%			29%	34%	25%		
Young [17]	Balanced	38 (19)	–	73%	27%	–	–	–	–	–	–	–	–
	Saline	39 (14)		79%	21%								
Ratanarat [18]	Balanced	–	–	–	–	–	–	–	–	–	–	–	–
	Saline												

*CKD* chronic kidney disease, *MV* mechanical ventilator, *ICU* intensive care unit, *SD* standard deviation, *IQR* interquartile range

## Outcome results

### Primary outcomes

**Fig. 3:**
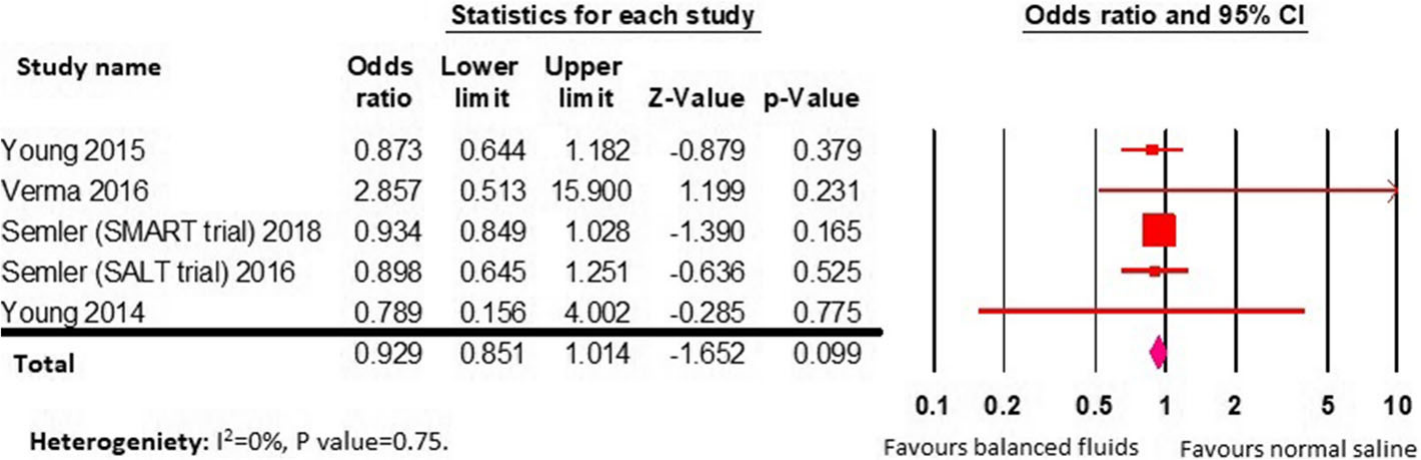
Forest plot for in-hospital mortality outcome

The incidence of in-hospital mortality in both groups were similar (11.5% vs 12.2%, OR 0.92; 95% CI 0.85–1.01; *P* = 0.09; *I*^2^ = 0%) (Fig. 3).

**Fig. 4:**
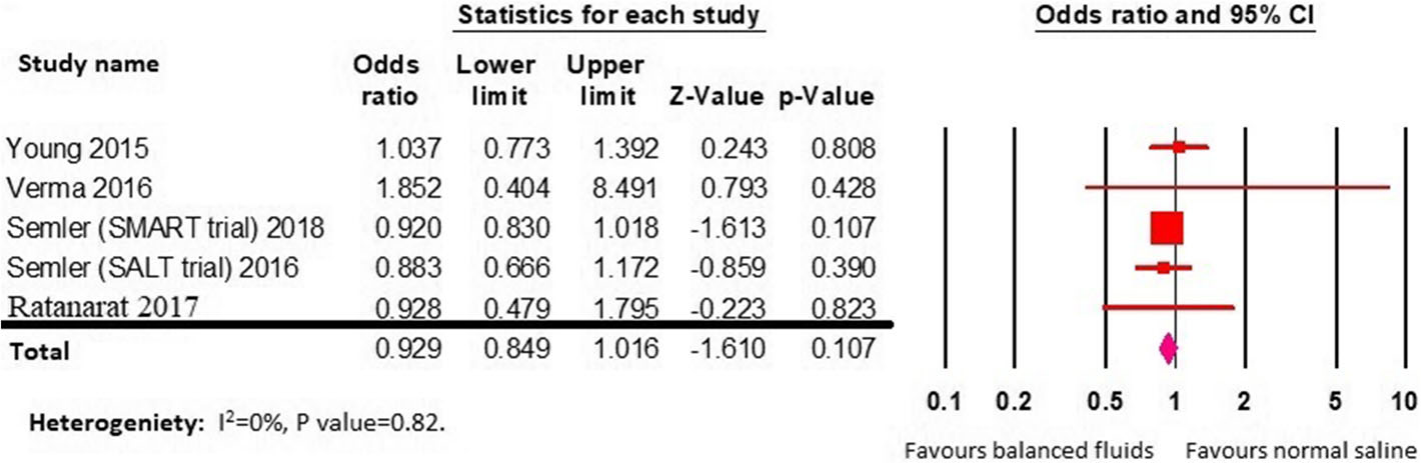
Forest plot for acute kidney injury (AKI)

As well, the difference in incidence of AKI was not statistically significant (12% versus 12.7%, OR 0.92; 95% CI 0.84–1.01; *P* = 0.1; *I*^2^ = 0%) (Fig. 4).

### Secondary outcomes

There was no significant difference between both groups of fluids in incidence of overall ICU mortality (OR 0.9, 95% CI 0.81–1.01, *P* = 0.08, *I*^2^ = 0%) or in need for new RRT (OR 0.92, 95% CI 0.67–1.28, *P* = 0.65, *I*^2^ = 38%) (Additional file 1: Figure S1 and Additional file 2: Figure S2).

## Discussion

Our meta-analysis revealed the following key findings: there are no significant differences between balanced crystalloids and isotonic saline, in the incidence of in-hospital mortality, AKI, ICU mortality, or need for new RRT in critically ill patients.

The strength of our meta-analysis is that it involved more than 19,000 patients from six RCTs. Retrospective studies and before and after treatment studies were excluded to decrease the risk of bias and confounding variables, as well; we included only studies that involved critically ill patients who received their management in the ICU to measure the response of this group of patients.

In two previously published meta-analyses evaluating the difference between both fluid types in unselected patient’s population involving surgical patients in perioperative period and kidney transplant in addition to critically ill patients, there was no difference in in-hospital mortality and incidence of AKI in both groups. Although both reviews have several limitations including the heterogeneity in patient’s population, and small sample size and few studies evaluated only critically ill patients, their results were in concordance with the results of our meta-analysis which was conducted exclusively in critically ill patients [13, 19].

A recently published randomized controlled trial (SMART study) involving about 15,000 patients concluded that balanced crystalloids have a decreased incidence of the primary outcome, major adverse kidney events defined in this study as composite outcome of death, persistent kidney dysfunction, and/or new RRT in comparison to isotonic fluids which is contrast to our analysis, but there was no difference between both fluid groups in each of the components of the primary outcome which included in-hospital mortality at 30 days, receipt of new RRT, and a final creatinine level more than 200% from baseline. As well, there was no difference in developing stage two or more kidney injury between both groups [15].

Our results are against the before and after treatment study in which Yunos et al. showed decreased incidence of AKI and new RRT when chloride liberal fluids were replaced by chloride restrictive fluids [20]. This study has several limitations that decreased the strength and the significance of its findings including that interventions were not randomized, and other fluid types were given during each study periods.

Furthermore, other studies did not show any difference in outcomes with the use of balanced crystalloid fluid solutions in comparison to normal saline. In two RCTs, there were no differences in AKI and in-hospital mortality when balanced fluids are compared to isotonic saline in heterogeneous ICU population [10, 11]. As well, these findings were evident in a retrospective study in non-surgical sepsis patients [5].

On the other hand, many retrospective studies showed improved outcomes when balanced crystalloids are compared to isotonic saline. Balanced fluids were associated with reduced in-hospital mortality and incidence of AKI in two retrospective analysis of large number of critically ill patients but both analyses have significant confounding variable that might have led to biases [21, 22].

This meta-analysis has proven that there is no difference between balanced crystalloids and isotonic saline solutions on the incidence of AKI, in-hospital mortality, overall ICU mortality, or new RRT in critically ill patients. The results of ongoing clinical trials are investigating the outcomes associated with different fluid types and will determine future recommendations in adjunct with this meta-analysis [23, 24].

## Limitations

Our meta-analysis has several limitations. First, some studies were of limited quality given the small sample size in comparison to other studies. Second, the patients in each study arm still could have received the other fluid type either before enrollment especially in the operation rooms or in the emergency department or during the study and this factor could alter our analysis findings. Third, the included studies have different designs and only three studies are double-blinded randomized controlled trials. Fourth, follow-up period was variable between studies as outlined in Table 1. Fifth, sensitivity analysis was not done since studies did not provide data about specific clinical situations like septic shock. Furthermore, we are limited by the fact that the results of meta-analysis were affected by one large randomized trial. Finally, study fluid administration was given only in first 24 and 72 h in two studies after randomization.

## Conclusion

There is no significant difference between balanced crystalloids and isotonic saline in the incidence of in-hospital mortality, AKI, ICU mortality, or need for new RRT in critically ill patients. Further powerful studies are required to determine the relationship between two fluid groups and various clinical outcomes.

## Additional files

Additional file 1: **Figure S1.** Forest plot of intensive care unit (ICU) mortality. (JPG 72 kb)

Additional file 2: **Figure S2.** Forest Plot for new renal replacement therapy. (JPG 92 kb)

## Abbreviations

AKI: Acute kidney injury
CI: Confidence interval
ICU: Intensive care unit
OR: Odds ratio
*P*: *P* value
RCT: Randomized clinical trial
RRT: Renal replacement therapy
