# Supplementary figures and images for: Balanced crystalloids versus isotonic saline in critically ill patients: systematic review and meta-analysis
Source: J Intensive Care. 2018 Aug 17;6:51. doi: 10.1186/s40560-018-0320-x (PMC6098635; doi:10.1186/s40560-018-0320-x)

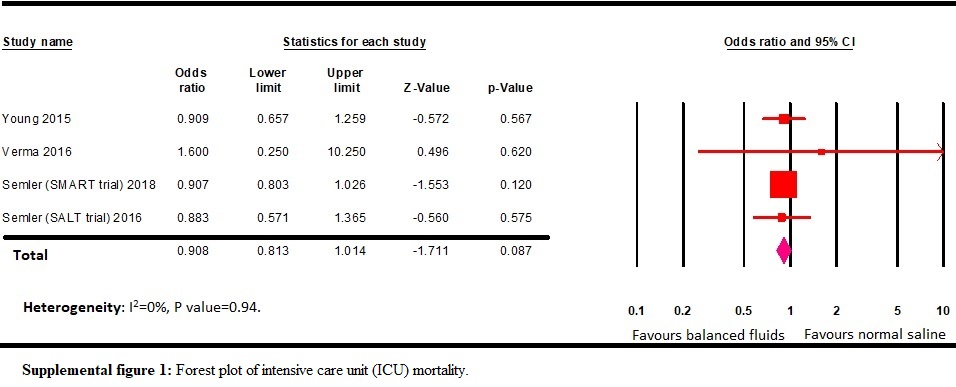

Supplement: Supplementary file 1 — Figure S1. Forest plot of intensive care unit (ICU) mortality. (JPG 72 kb) [file 40560_2018_320_MOESM1_ESM.jpg]

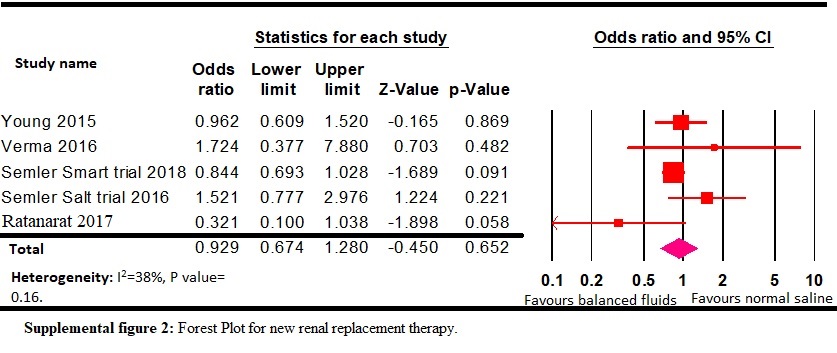

Supplement: Supplementary file 2 — Figure S2. Forest Plot for new renal replacement therapy. (JPG 92 kb) [file 40560_2018_320_MOESM2_ESM.jpg]
